# Trace Minerals Supplementation with Great Impact on Beef Cattle Immunity and Health

**DOI:** 10.3390/ani12202839

**Published:** 2022-10-19

**Authors:** Roberto A. Palomares

**Affiliations:** Group for Reproduction in Animals, Vaccinology & Infectious Diseases (GRAVID™), Department of Population Health, College of Veterinary Medicine, University of Georgia, 2200 College Station Rd, Athens, GA 30602, USA; palomnr@uga.edu

**Keywords:** trace minerals, supplementation, beef cattle, health, immunity

## Abstract

**Simple Summary:**

Supplementation with trace minerals (TM) is a husbandry strategy to improve cattle health. There is solid evidence of the beneficial effects of TM supplementation on the immune system. The concentration of TM in the soil is variable across the USA, with several regions having deficient levels in forages. Therefore, TM supplementation is highly recommended especially in areas where forages do not supply the mineral requirements. Before starting TM supplementation, it is important to evaluate the herd’s mineral profile, and the amount of TM the animals are consuming. Oral free-choice TM may not be sufficient to satisfy the requirements in certain situations, and could lead to TM deficiencies. This is due to a high variability in TM composition and intake, binding to undigested feed particles, reduced absorption, and antagonisms. Single, oral pulse-dose supplementation provides a controlled and homogeneous amount of TM intended to remove such a variation. However, this strategy does not efficiently increase circulating and hepatic TM levels. Parenteral TM supplementation has resulted in a more efficient increase in TM concentration. The strategic supplementation combining injectable TM during critical times of cattle management (e.g., vaccination) in conjunction with oral free-choice supplements has shown significant benefits for the immune response and protection against respiratory disease in beef cattle, reducing morbidity and treatment costs.

**Abstract:**

Trace minerals (TM) play an important role in cattle immunity, health and performance. Although TM are needed in small quantities, they are fundamental for enzymes involved in antioxidant protection against cellular damage and several pathways of the immune response. Cattle TM status results from the balance between TM dietary intake and their requirements. Free-choice oral TM supplementation is a common practice in beef cattle production systems. However, there is a high variation in TM intake and thus TM status and bioavailability in animals receiving free-choice oral TM supplements. Strategic pulse-dose supplementation during critical points of beef cattle management provides a controlled amount of TM intended to remove such a variation. Adequate TM supplementation should not only satisfy the basal requirements but also provide a source of TM when there is a higher demand of the antioxidant systems or during the development of the immune response. This paper reviews the research-based evidence of the effects of TM supplementation on immunity and its impact on beef cattle health. This review highlights the benefits of a novel approach of strategic administration of injectable trace minerals (Se, Zn, Cu and Mn) during critical episodes of cattle management (e.g., around weaning or at vaccination) in combination with free-choice oral supplementation to maintain adequate TM and oxidative status, enhanced immunity and overall cattle health. This strategy has proven to decrease morbidity, which would positively impact the productivity of the beef cattle systems.

## 1. Introduction

Beef cattle operations from cow/calf farms to feedlot systems achieve their production goals by ensuring the health of the animals under their care. Of the health issues that can interfere with reaching these goals, bovine respiratory disease (BRD) is the most prevalent. Multiple factors, such as suboptimal nutrition, stress related to weaning, transportation, commingling, environmental extremes, and high stocking density contribute to the occurrence of BRD [1]. Therefore, an effective beef cattle health program should integrate approaches designed to offer adequate nutrient balance (quality and quantity), minimize stress, reduce the level of exposure to pathogens (through adequate biosecurity and biocontainment), generate protective immunity through colostrum and vaccination, identify and isolate sick animals, and identify the associated pathogens.

Balanced nutrition that includes adequate supply of protein, energy, vitamins and minerals is one of the pillars of achieving optimal health in beef cattle operations. Trace minerals (TM) play critical roles in the immune system’s response to potential pathogens, which will be the focus of this review. As a result, TM are important for the cattle health and performance [2]. Although these elements are needed in small quantities, they are fundamental for many biological processes. Examples of these processes include energy production, signal transduction, nucleic acid replication, transcription and translation, and antioxidant protection against cellular damage. Because TM have specific functions in the animal’s innate and acquired immune responses [3], adequate TM supplementation is crucial for the development of protective immunity, especially in stressed animals. Four TM, namely Se, Cu, Zn, and Mn, exert specific positive effects on the immune response to different pathogens. In addition, Cr is known to be beneficial for the health of cattle during periods of stress by reducing cortisol levels and modulating insulin and glucose concentrations. As a result, these elements are currently included in commercial TM formulations.

Numerous factors influence the TM status of cattle, including the mineral content of soils, forages, and feedstuffs, cattle age and production stage, TM requirements and dry matter intake (DMI), presence of antagonists in feed, water, and forages and the type of supplement formulations. Supplementation with TM can be achieved using either oral (e.g., powder formulations, salt blocks, drench and rumen boluses) or injectable routes of administration. It is important to recognize the differences in the bioavailability of the TM between these administration routes. Clinical and scientific evidence supports TM supplementation in beef cattle herds and growing steers. Numerous studies have shown the effects of TM on the cattle immune response as well as their health and performance [4,5,6,7,8,9,10,11,12,13,14,15,16,17,18,19,20,21,22,23,24,25]. However, many of those studies differ substantially in their experimental designs, protocols, TM formulations, baseline diets, and outcomes. Consequently, there is considerable variation in their conclusions. In addition, the majority of the studies did not objectively assess the effects of TM on the cell-mediated immune response to specific pathogens affecting cattle (e.g., antigen recall leukocyte proliferation or proportion of lymphocyte subsets after challenge). The present manuscript reviews the research-based evidence of the effects of supplementation with Se, Cu, Zn, Mn and Cr on the immune response and health of beef cattle. This review highlights the effects of strategic supplementation based on injectable TM during critical times of cattle management (e.g., vaccination) in conjunction with oral free-choice supplements, on the humoral and cell-mediated immune responses and protection against respiratory disease pathogens in beef cattle, which is a relatively new area of investigation and the author’s main research focus.

## 2. Beef Cattle Health Program: Basic Principles

The overall health of cattle depends on an appropriately functioning immune system, its interaction with the environment (e.g., climate, nutrition, management, cattle density), exposure to potential pathogens, and their virulence. In most instances, the level of exposure to pathogens is determined by hygienic conditions and biosecurity. Health is achieved when the animal’s level of immunity exceeds the disease challenge. Consequently, the goal of all beef cattle health programs is to simultaneously increase the level of immunity while reducing pathogen exposure [26]. Several factors can increase a herd’s susceptibility to disease, including poor hygiene and biosecurity, low immuno-competence due to lack or ineffective vaccination, stress, inadequate management, nutritional deficiencies, high population density, and failure in passive transfer, among others [26]. Therefore, the health of the cattle can be improved through preventative management strategies focused on optimizing nutrition, enhancing colostrum quality, minimizing stress, improving biosecurity, and safe and efficacious vaccination procedures. Appropriate nutrient supply, including supplementation with key minerals (e.g., Cu, Se, Mn, and Zn) is a key component in cattle health programs as it provides the foundation for other health management strategies (e.g., vaccination).

Adequate maternal antibody transfer [e.g., Immunoglobulin G (IgG) serum concentration >1600 mg/dL] is the first line of defense for the newborn calf [27]. Inadequate passive transfer occurs in 10–25% of newborn beef calves [28]. Satisfactory antibody passive transfer is achieved when newborn calves absorb enough immunoglobulins (200–400 g) after suckling high quality colostrum (>50 g IgG/L) from their dams within the first six hours of life [28]. Thus, it is vital to increase the colostrum immunoglobulin concentration through effective vaccination (e.g., BRD, Clostridium complex, and pinkeye) of female cattle, especially in the last third of pregnancy. Not only does vaccination increase the concentration of antibodies, it also increases the number of immunologically relevant cells (lymphocytes) and concentration of interferons in colostrum [29]. It also is important for cows to maintain an adequate body condition score (6–7, on a scale from 1 to 9, with 1 being severely emaciated and 9 extremely obese) and mineral status before calving to enhance IgG concentration. In beef cattle, marginal TM deficiencies negatively affect antibody production after vaccination, as well as colostrum quality and quantity, and subsequent passive antibody transfer to the calves [30].

Vaccination of calves before or after weaning is a major tool to prevent the many diseases affecting beef cattle, such as BRD. However, vaccines are not 100% efficacious, as the subsequent level of protection depends on many factors. Low response to BRD vaccination may occur as the result of several factors, including nutritional deficiencies, poor management, high level of stress, concurrent diseases, young age at vaccination, high levels of maternal antibodies, and genetics [31]. Mineral deficiencies have been identified as an important risk factor contributing to the development of BRD in nursing calves [32]. Additionally, many bovine practitioners are concerned that marginal deficiencies in Se, Cu, and Zn are responsible for the failure of some animals to respond appropriately to vaccination against BRD.

It is critical for cattle to develop sufficient immunity before encountering stressful situations or being exposed to pathogens. For example, management practices (e.g., castration, weaning, transportation, and commingling) can affect their ability to produce an adequate immune response after vaccination [33]. During stressful periods, animals undergo an increased metabolic demand that results in oxidative stress. This involves an imbalance between the production and neutralization of pro-oxidant substances known as reactive oxygen species (ROS). These free radicals cause significant cell damage by peroxidation of lipid cell membranes [34] to which leukocytes are particularly sensitive. This impairs the immune function and increases the animal’s susceptibility to disease. For this reason, stressed cattle are more prone to BRD shortly after arrival at the feed yard than preconditioned cattle, which experience lower levels of stress [35].

Importantly, Cu, Zn, Se and Mn are structural components of the enzymes that neutralize ROS, and strategic supplementation with TM can minimize the negative effects of stress on the immune system. Supplementation with TM has been suggested as a husbandry strategy to improve the immune response to vaccination (especially against respiratory viruses) and the overall health status of cattle. Therefore, it should be considered as an essential component of beef cattle health programs.

## 3. Cattle Immunity

**Figure 1:**
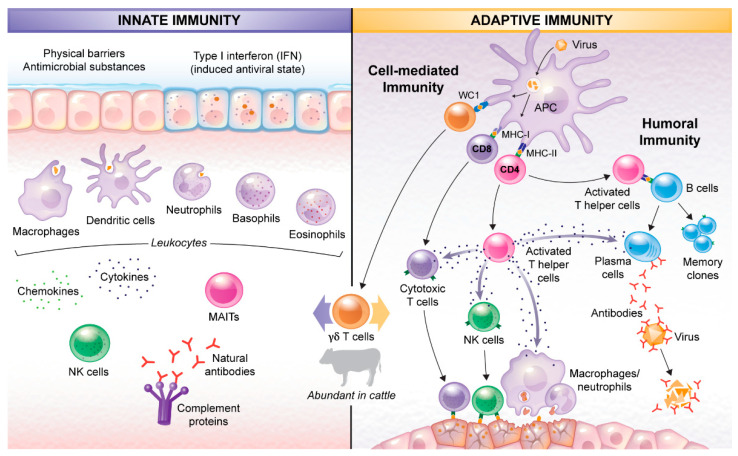
The bovine immune system. The innate immunity mainly involves epithelial barriers, phagocytic cells and their chemokines and cytokines, Natural killer (NK) cells, Mucosa-associated invariant T cells (MAIT), Type I interferon and Complement proteins. The adaptive immunity consists of antibody production by Plasma cells (differentiated B lymphocytes) and cell-mediated immunity handled by T lymphocytes. Gamma delta T cells are the most abundant T lymphocytes in cattle and play important roles in both innate and adaptive immune responses.

The immune system has two main components: innate and adaptive immunity. The innate immunity is the first line of protection against pathogens. It includes physical barriers (dermal and mucosal epithelia), antimicrobial substances produced by epithelial cells, the complement proteins, and phagocytic cells (e.g., neutrophils, macrophages and dendritic cells) and their cytokines and chemokines. Furthermore, the innate immunity includes the type I interferon (IFN)-induced antiviral state, gamma delta T cells (γδ T), mucosa-associated invariant T cells, non-phagocytic granulocytes (e.g., eosinophils and basophils) and natural killer cells [36] (Figure 1). Neutrophils identify infectious agents by recognition of pathogen-associated molecular patterns using Toll-like receptors [37,38]. After pathogen recognition, phagocytic cells activate and initiate phagocytosis and killing of the engulfed microorganisms. Importantly, the innate immune system is not pathogen-specific and responds to different antigens in a similar manner. Moreover, this component of the immune system does not produce a memory or anamnestic response.

The adaptive immune response is initiated after vaccination or natural exposure to foreign agents. This response consists of humoral (antibody production by B cells) and cell-mediated immunity (handled by T lymphocytes and their cytokines) [36]. This part of the immune system is pathogen-specific, requiring recognition of the pathogen, activation of T and B lymphocytes, differentiation into effector and memory cells, and repair of damaged tissues (Figure 1). This branch of the immune system creates memory that subsequently responds to repeated exposure to the same pathogen.

B lymphocytes mature in the bone marrow and are released into blood where they circulate and populate lymphoid tissues. Plasma cells, which are differentiated effector B cells, function as the main component of the humoral immune response, producing specific antibodies after vaccination or antigen exposure [36]. Antibodies are Y-shaped proteins having different isotopes (e.g., IgG, IgM, IgD, IgA and IgE) and specific roles, including neutralization, complement activation, opsonization and phagocytosis, and antibody-dependent cellular cytotoxicity [36,39]. B cells also recognize foreign antigens and present them to T lymphocytes, which further stimulate greater antibody production by plasma cells. In addition, B cells located in the mucosa secrete IgA antibodies as part of the humoral response. The IgA antibodies help neutralize antigens and prevent attachment of pathogens to the epithelial cells, thus preventing the pathogens from colonizing the main routes of entrance and underlying tissues.

T lymphocytes are produced in the bone marrow and mature in the thymus. They are categorized as helper, cytotoxic, and gamma-delta T cells. T helper cells are characterized by the CD4 protein that collects information from antigen presenting cells expressing the major histocompatibility complex class II (MHC-II) [40,41]. One of the important roles of T helper cells is to release cytokines (e.g., IFN-γ). These proteins stimulate a variety of immune cells, including neutrophils, macrophages, additional T helper cells, cytotoxic T cells and B lymphocytes as part of the development of the adaptive immune response [42]. For instance, production of antibodies by plasma cells involves the secretion of interleukins-2 (IL-2) IL-4 and IFN-γ by CD4^+^ T cells.

In contrast, cytotoxic T cells express CD8 through which they recognize and destroy virus-infected cells via MHC-I [43]. Gamma–delta T cells, which express the surface protein WC1, comprise the most abundant subset of T cells in cattle and have the ability to activate independently of the MHC proteins. They are present in high numbers in epithelial sites, haemal lymph nodes, and the thymus [44]. These cells play wide-ranging roles in both innate and adaptive immune responses, including immune regulation, cytokine production, and local defenses at the mucosal epithelium, [45].

## 4. Role of Trace Minerals in Bovine Immunity

**Figure 2:**
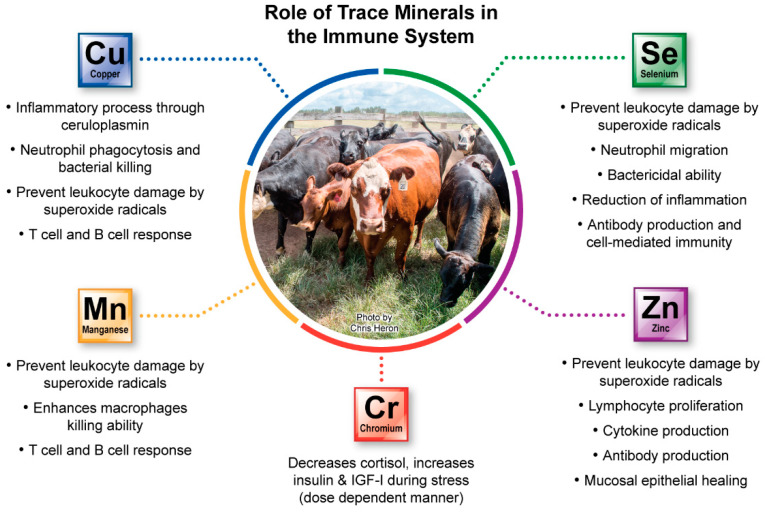
The main roles of trace minerals (TM) in the bovine immune system. Copper, Mn and Zn are structural and functional components of the antioxidant enzyme superoxide dismutase (SOD) and Se is a component of the enzyme glutathione peroxidase (GPx). These enzymes protect leukocytes against membrane damage by free radicals. These TM play important functions mainly in epithelial integrity, leukocyte migration, phagocytosis and bacteria killing, cytokine production, antibody secretion and cell-mediated immunity (CMI).

The main roles of Zn, Cu, Se, Mn and Cr in the bovine immune system are summarized in Figure 2.

### 4.1. Zinc

Zinc is an important structural and functional element of several enzymes and proteins required in different metabolic pathways [46]. Zinc plays an essential role in tissues that undergo mitosis (e.g., lymphoid tissue, skin, testicular seminiferous tubules, and mucous membranes). In doing so, Zn contributes to the strength of the mucosal lining of the respiratory and digestive systems. It also is involved in mucosal tissue repair and epithelial integrity. Its ability to affect proliferation of epithelial cells is due to its role in nucleic acid replication (e.g., DP1-DP2 zinc fingers, ribonucleotide reductases), transcription (e.g., RNA polymerases, aminoacyl-tRNA synthetases and transcription factors), and protein synthesis via mechanistic target of rapamycin kinase, mTOR [46]. Zinc is essential for clonal expansion during lymphocyte proliferation and differentiation before effector functions are executed [47,48,49]. In addition, its role in mRNA expression and subsequent translation into proteins makes it critical for cytokine production by macrophages and lymphocytes during both innate and acquired immunity [50].

Zinc is a powerful antioxidant as a structural component of superoxide dismutase (Zn-SOD), a cytosolic and mitochondrial intermembrane enzyme involved in conversion of superoxide radicals (O_2_^−^) into hydrogen peroxide (H_2_ O_2_) [51]. Zinc also plays an important role in the synthesis of nitric oxide by macrophages [52], which is an essential molecule for bacteria killing.

As might be anticipated, Zn deficiency impairs both branches of the immune response. In the innate immunity, Zn deficiency negatively affects neutrophil and macrophage migration, phagocytosis and killing of microbes, as well as dermal and mucosal integrity. In the adaptive immunity, Zn deficiency affects thymic function as well as activation, proliferation, differentiation and cytokine secretion by lymphocytes [53]. Zinc status has been associated with the risk of neonatal diarrhea in calves [54]. Further, serum Zn concentrations have been negatively correlated with abortion rates [55].

### 4.2. Copper

Copper is required for various enzymes and proteins involved in mitochondrial energy production and other metabolic pathways [48]. Its primary role in immunity is through its involvement in the enzyme Cu-SOD, a metalloprotein responsible for production of hydrogen peroxide radicals by neutrophils [56]. These free radicals are used to generate powerful oxidizing agents such as hydroxyl radicals by the myeloperoxidase system [46,49]. Cu-SOD is responsible for maintaining a balance in the production of free radicals needed to kill bacteria via the oxidative burst activity. Furthermore, this enzyme is important in mitigating oxidative stress as it scavenges superoxide radicals. In doing so, Cu-SOD prevents damage to leukocyte membranes by avoiding excessive accumulation of superoxide free radicals. Copper also is a component of ceruloplasmin, an acute phase protein with a prominent role in bovine immunity [57], especially in stressed animals. In this regard, concentrations of ceruloplasmin have been used as a biomarker to evaluate Cu status and the effects of TM supplementation in cattle [58].

Copper deficiency may occur as a consequence of low Cu dietary levels or consumption of molybdenum-rich pastures [58]. Similarly, excessive sulfur in cattle diets (e.g., high sulfate water or feeding ethanol coproducts such as distillers grains) negatively affects Cu absorption. In the rumen, S can interact with molybdate to form thiomolybdate complexes which have very high affinity for Cu, negatively affecting its absorption and bioavailability, resulting in Cu deficiency [59]. Copper deficiency is associated with impaired neutrophil phagocytic function [57] and affects the acute phase protein response. However, Arthington et al. [60] showed that Cu deficiency in crossbred beef heifers did not affect in vitro or in vivo neutrophil chemotaxis after injection of an inflammation-inducing compound. In another study, Arthington et al. [58] observed that calves with molybdenum-induced Cu deficiency did not elevate plasma ceruloplasmin concentrations after BHV1 challenge, but had a significant increase of haptoglobin and fibrinogen concentrations compared with calves with normal Cu status. Overall, Cu deficiency has been associated with poor calf health and performance (mainly diarrhea, growth retardation and mortality) [54], likely through reduced innate immunity, failure of passive transfer, decreased antibody production and impaired cell-mediated immunity [61]. Severe Cu deficiency in cattle may result in sudden death due to heart failure [62].

### 4.3. Selenium

Selenium is a structural component of the enzyme glutathione peroxidase (GPx) which catalyzes the reduction of hydrogen peroxide to water and oxygen. This enzyme is important to providing nicotinamide adenine dinucleotide phosphate (NADPH) for the respiratory burst along with glutathione reductase, and to maintaining the bactericidal capacity of neutrophils [63,64]. In addition, this enzyme inactivates ROS production, preventing subsequent leukocyte damage [63].

Selenium plays a major role in neutrophil migration into tissues and subsequent inflammation [63]. Selenium deficiencies have been associated with decreased neutrophil migration and killing activity [65], reduced GPx antioxidant functions, and impaired B-cell function and antibody production. Se-deficient calves had reduced antibody production 14 d after BHV1 challenge [66]. However, in that study, Se deficiency did not affect DMI or rectal temperatures [66].

Selenium deficiency causes multiple health problems. Se-deficient calves may have muscular degeneration known as White muscle disease. This condition may result in immediate death due to myocardial degeneration. Marginally Se-deficient calves may display weakness and low vigor. A retrospective study in France indicated that deficient and marginal Se status of cows was associated with increased risk of infectious abortion, high calf morbidity and mortality due to poor immune response [54], which may be related to altered iodine metabolism [67]. Further, Se and vitamin E deficiency in cows have been associated with increased incidence of retained fetal membranes [54].

### 4.4. Manganese

Manganese is a component of several enzymes (e.g., arginase, pyruvate carboxylase, and SOD). It also functions as an enzyme activator for various hydrolases, kinases, and transferases [68]. Evidence of the roles of Mn in the immune response is limited. Manganese has an essential function in scavenging ROS produced by phagocytic cells since it is a structural component of the Mn-SOD enzyme [69]. Dietary manganese deficiency has relatively mild effects on immunity, with the exception of impaired humoral immune response [70]. Manganese deficiency (plasma Mn < 76 nmol/L) has been associated with abortion, long bone congenital deformities (including enlarged joints, stiffness, twisted legs, shorter bones, as well as general physical weakness) in newborn calves [71,72] and tongue neuromuscular disorder in cows [73].

### 4.5. Chromium

Chromium is essential for the immune response during stress, which is particularly important in feedlot cattle. Chromium is required to promote the action of insulin and insulin-like growth factor I in tissues such as muscle and liver. Thus, it is implicated in different metabolic pathways (e.g., glucose tolerance and clearance rate) related to growth and homeostasis [74]. Moreover, Cr appears to decrease circulating cortisol concentrations during highly stressful situations [7]. Chromium affects several pathways of the immune system. The effects of Cr on lymphocytes show a biphasic pattern with a stimulatory effect at low concentrations but an inhibitory effect at high concentrations [75]. Chromium reduced the levels of the immunomodulatory cytokines IL-2, IFN-γ, and TNF-α following in vitro mitogen stimulation in lactating Holstein cows [76]. In addition, Cr stimulated lymphocyte proliferation after mitogen stimulation [77], cell-mediated immunity after injection of phyto-hemagglutinin in young calves [78], as well as antibody production following immunization with infectious bovine rhinotracheitis virus [77] and tetanus toxoid in cattle [79].

## 5. Trace Mineral Status in Beef Cattle

In general, the TM status results from a balance between dietary TM intake and requirements. Moreover, the TM status is affected by the intestinal absorption, chemical interactions, and mineral antagonisms in the gastrointestinal tract. Liver samples to determine hepatic TM concentrations represent the gold standard and the best choice for determining the TM status in cattle. Liver is the organ where most TM of interest in cattle are stored, incorporated into enzymes and then released when needed. Blood TM concentrations may be variable, inconsistent and have low predictive value of the real TM status. Assessment of the TM status of beef herds is used to determine whether a nutrient deficiency exists (marginal or clinically evident), to assess the prevalence of a deficiency, or to estimate the endogenous reserves of TM [80]. Copper levels in liver samples should be above 25–50 ppm to be considered normal (range: 50–600 μg/g; on a dry matter (DM) basis, Table 1) [81]. Liver levels of Se of 0.8–1.0 ppm (range: 0.7–2.5 μg/g; [81]) are considered to be adequate, and levels below 0.2 ppm are considered to be critically deficient [80]. Liver Mn concentrations below 9 ppm (range: 5–15 μg/g [81]) can be considered an indication of marginal deficiency. Hepatic Zn concentrations under 80 ppm can be considered marginal or deficient [80] (range: 90–400 μg/g [81], Table 1). Nevertheless, liver tissue is not an ideal sample for accurate determination of Zn status, probably due to its particular bioavailability, dynamic chemical interactions and extensive use throughout the body. Muscle and bone tissues represent the main Zn pools. However, sampling these tissues is not a practical procedure to be performed in the field.

Several factors affect the TM status of cattle, including mineral content of soils, forages and feedstuffs, high variability of mineral intake, presence of mineral antagonists in water, forages, and feedstuffs, and variable mineral requirements. The requirements are the guidelines to determine the animal’s needs and formulate TM supplements. Trace minerals requirements derive from refereed research summarized by the National Academies of Sciences, Engineering, and Medicine (NASEM), Board on Agriculture Subcommittee on Beef Cattle Nutrition [82] (Table 1). Trace mineral requirements vary and may be affected by several factors including cattle biological type, breed, physiological state, health and nutritional status, management practice, and environmental conditions, among others.

Concentration of TM in the soil is variable across the USA, several regions containing deficient levels of Se, Zn, Co, and Cu in forages [83]. Therefore, TM supplementation is highly recommended in those areas where forages do not supply the mineral requirements [84]. A study performed by Kansas State University and USDA on 352 forage samples from cow/calf operations in 18 USA states revealed deficiency of important TM in a high proportion of samples [80]. Only 2.5% of the forage samples contained adequate zinc levels. Copper and cobalt levels were adequate in 36% and 34.1% of the samples, respectively [80]. In addition, 49.7% of the samples were considered marginal for Cu, and 14.2% of the samples were classified as critically deficient. Another report of 23 USA states showed similar results where only 4%, 15% and 26% of fescue samples from Midwest USA had adequate levels of Se, Zn and Cu, respectively [85]. Moreover, only 18, 24 and 24% of native forage samples were adequate in Cu, Se and Zn, respectively [85]. In both surveys, high percentage of the samples had very high (8–13%) or marginally high (17–48%) levels of iron, molybdenum and sulfur [80,85], which results in Cu deficiency due to antagonistic effects limiting Cu absorption through the gastrointestinal tract. The TM status of cattle is highly dependent on mineral intake by the animal, which is affected by stress, the animal’s physiologic status, the feeding protocol and the type of diet. For instance, DMI decreases after arrival at the feedyard, where a high proportion of calves do not eat sufficiently during the first 48–72 h. In general, stressed calves reduce their feed intake to an average of 1.5% of body weight during the first two weeks after arrival [86]. This deprives the immune system of the nutrients needed to generate an effective response. Short-term reduced intake and transient deficiency of Mn, Cu, Zn, and Se will probably not induce an immediate clinical deficiency response in cases where prior intake was adequate. However, a chronic insufficient TM intake for several weeks may lead to depletion of the body stores, resulting in altered metabolism, impaired immunity, and poor health status and production performance. The rate of tissue (e.g., liver) depletion for different TM in cattle (and the switch to clinical deficiency) is not completely understood. Deficiency of TM progressively affects different physiologic and metabolic functions. A marginal deficiency in TM is not visually evident, and borderline levels may continue to decrease over time, affecting the immune response, which makes the animals more susceptible to common respiratory pathogens. A subclinical TM deficiency eventually may become evident at the herd level, with increased morbidity and reduced growth and reproductive performance [87]. A clinical TM deficiency occurs when TM levels are so low that they result in specific clinical signs of disease (e.g., stiffness and muscle weakness due to muscle degeneration known as White muscle disease, caused by severe Se deficiency). It is highly recommended to always evaluate the herd’s mineral profile, the amount of TM that the animals may be consuming, and therefore determine the TM formulation the animals may need.

## 6. Oral Trace Minerals Supplementation, Effects on Beef Cattle Health

Oral TM supplementation is a basic management practice in beef cattle operations. There are different sources of trace minerals to supplement cattle depending on their organic or inorganic composition. Organic sources (e.g., chelates, amino acid or polysaccharide complexes, propionates, proteinates and yeast derivatives) are more biologically available to the animal. Thus, organic sources may be beneficial at periods of high stress and low feed intake [88] and have been reported to offer benefits on health and performance compared with the inorganic forms [24,89]. Inorganic forms of TM (e.g., sulfates, oxides, carbonates, and chlorides) are structured with molecules of sulfur, oxygen, and chloride, among others. The most bioavailable inorganic source of TM is generally the sulfate forms, followed by the chloride forms of Zn, Cu, and Mn. In contrast, the oxide inorganic form is the least bioavailable and should be avoided for beef cattle feeding programs. Oral mineral supplements can be provided as free-choice mixture of mineralized salt, blocks or in combination with energy and/or protein supplements in the total mixed ration.

The effects of oral TM supplementation on health and disease resistance have been assessed by providing single or combined TM compounds. Studies using TM combinations provide limited information about the specific effects of TM on the immune response and health. Thus, this section of the manuscript focuses on the effects of oral supplementation with individual TM on cattle health and immunity. During the last decades, numerous studies have demonstrated the benefits of oral TM supplementation on beef cattle immunity, health and performance [4,5,6,7,8,9,10,11,12,13,14,15,16,17,18,19,20,21,22,23,24,25]. However, other studies have shown marginal or insignificant effects of oral TM supplementation on beef cattle metabolism, growth, immunity and health [90,91,92,93,94,95,96,97,98,99,100,101,102,103,104,105,106,107,108]. Many of these studies have provided inconsistent results due to differences in mineral forms supplemented (organic versus inorganic), high variability in TM concentration in control diets, the animals’ TM requirements and initial TM status, variation in animal intake and absorption, chemical mineral interactions and antagonisms, level of stress, and differences in experimental models and endpoints. These factors have made interpretation and comparison of data from different studies extremely difficult. Further, in some of these studies, the reported effects of TM on the immune function and protection may not be clinically relevant or influence the cattle health status. Thus, this review will mainly discuss studies assessing the effects of oral supplementation with individual TM on important immunological or clinical outcomes for beef cattle production systems.

### 6.1. Oral Zinc Supplementation

According to the NASEM [82], the recommended Zn dietary level is 30 mg/kg DM (Table 1) [82]. Multiple studies have demonstrated the effects of Zn supplementation on the humoral and cell-mediated immune responses and health [8,10,11,12,13,92]. Supplementation of steers with Zn-methionine resulted in greater DMI and improved health status after inoculation of BHV1 compared with non-supplemented steers or animals receiving supplementation with ZnO [13,109], Zinc proteinate (71 mg/kg DM) or Zn-SO_4_ (67 mg/kg DM, [110]). In addition, steers supplemented with Zn-methionine or ZnO (25 mg of supplemental Zn/kg DM) had greater BHV1-specific antibody titers compared with steers receiving a control diet (26 mg of Zn/kg DM) [6]. Moreover, increasing the amount of supplemented Zn-methionine (from 35 to 70 mg/kg DM) in beef calves receiving a 65% concentrate basal diet after weaning and transportation significantly reduced the incidence of BRD (23.6 and 9.7% for low and high Zn-methionine, respectively) during a 28-day receiving period and a 21-day step-up period [92].

In a subsequent report, supplementation of grazing Angus crossbred heifers with ZnSO_4_ (360 mg of Zn/d), or Zn amino acid complex (360 mg of Zn/d) after transportation tended to enhance cell-mediated immune response to phyto-hemagglutinin injection compared with non-supplemented heifers [12]. Studies showing the effects of feeding Zn-deficient-diets (e.g., 17 mg/kg DM) or abrupt Zn depletion in beef cattle revealed a significant reduction in skin cell-mediated immune response to phyto-hemagglutinin [111,112]. Moreover, Zn supplementation has been demonstrated to enhance hoof quality scores [113], while Zn deficiency has been previously associated with lameness and hoof deformation [114]. Specifically, supplementation of grazing calves and finishing steers with Zn-methionine decreased the incidence of foot rot compared with non-supplemented animals [8], reflecting the importance of Zn for maintenance of the healthy tegumentary tissues. In contrast, other studies have not shown positive effects of different sources of oral Zn supplementation (methionine, sulfate, and propionate) on beef cattle immunity [93,102], health and performance [100,102].

### 6.2. Oral Copper Supplementation

According to NASEM [82], finishing steers and beef cows require 10 mg Cu/kg DM [82]. When forage samples contain less than 8 ppm of copper, they are considered deficient. The effects of oral Cu supplementation on animal health and immunity depends on the animal’s physiologic conditions, immune competence and mineral status. Copper supplementation of beef cows resulted in improved health status and increased growth performance of their calves [115]. In addition, Cu supplementation of weaned calves enhanced their oxidative status [22], immune response, protection and overall health [92,116].

Supplementation of weaned beef calves with Cu-lysine (16 mg of Cu/kg DM) resulted in increased plasma Cu concentration, enhanced phagocytic and oxidative burst activities of monocytes compared with control calves fed a basal diet of corn silage/soybean meal with no supplemental Cu (7 mg of Cu/kg DM) [117]. In addition, Galyean et al. [92] reported that Cu-lysine supplementation (5 mg of added Cu/kg) of beef steers was associated with a reduction (not statistically significant) in BRD morbidity (13.9%) compared with the control animals (20.1% morbidity) receiving a baseline diet that provided 3.25 mg of Cu/kg from CuO. However, Cu-lysine-supplemented animals had lower average daily gain (ADG) and DMI. Dorton et al. [118] observed that Cu supplementation of Angus steers (10 or 20 mg Cu/kg DM) tended to increase the antibody response compared with animals receiving a control diet (7 mg Cu/kg DM).

Molybdenum, sulfur and iron are Cu antagonists. High levels of Mo (>1–3 ppm) in forages and feedstuffs (or when the Cu:Mo ratio is <3:1) may negatively impact Cu status, resulting in Cu deficiency-like effects. Calves fed high levels of Mo (5 mg of Mo/kg DM of diet) showed reduced Cu-SOD activity, ceruloplasmin concentration and TNF-α production by monocytes after BHV1 inoculation compared with Cu-supplemented calves (10 mg Cu/kg DM), which had adequate Cu status [116]. In the same study, the calves supplemented with Cu and inoculated with BHV1 and *Pasteurella* two days after weaning had greater body temperature and TNF-α concentration after challenge, which was attributed to improved immune response and greater cytokine production [116]. Poor neutrophil phagocytic activity was observed in Cu-deficient cattle (induced by feeding Fe or Mo) compared with cattle supplemented with adequate amounts of Cu in the diet [119].

Other studies failed to demonstrate positive effects of Cu supplementation on beef cattle health, or had inconsistent results [47,96,97,100,104,115]. Stressed calves supplemented with Cu (5 mg/kg DM for 133 d) had increased antibody production (to pig red blood cells) and cell-mediated immune response (to dinitrochlorobenzene stimulation) [120]. However, these effects were not observed in unstressed calves [120]. Copper source (Polysaccharide complex of copper SQM^®^ Cu or CuSO_4_) did not affect growth performance and BRD morbidity in newly received heifers, but heifers receiving CuSO_4_ had greater antigen-specific antibody titers than did heifers on the SQM^®^ Cu treatment on days 14 and 21 [100]. In steers with adequate Cu status, supplementation with Cu (5 mg/kg DM as Cu sulfate or Cu lysine) did not affect antibody production, cell-mediated immunity or growth performance [121]. Similarly, Cu supplementation before and after calving (either inorganic, organic, or organic with extra Zn) of 2-year-old crossbred beef cows having Cu hepatic borderline levels (~50 mg/kg) did not improve pregnancy rates, maternal antibody transfer or calf health when compared with non-supplemented cows [96].

### 6.3. Oral Selenium Supplementation

According to NASEM [82], finishing steers and beef cows require 0.10 mg Se/kg DM [82]. In general, oral Se supplementation has been documented to enhance both humoral and cell-mediated immune responses [56]. However, similar to other reported mineral assays, assessing the effects of oral Se supplementation on cattle health has been challenging, and provided inconsistent conclusions.

Beef cows supplemented with 120 mg of Se/kg of salt-mineral mix had greater colostrum IgG concentration than Se-deficient cows. In addition, calves born to Se-supplemented cows had higher post-suckle serum concentrations of IgG than calves born to control cows [5]. In another report, feeding Se-biofortified alfalfa hay to pregnant cows resulted in increased colostrum Se and IgG1 concentrations but did not affect short-term serum antibody concentration in their calves [108]. Hall et al. [18,19] reported that weaned calves that had been fed Se-enriched alfalfa hay (0.95, 1.55, or 3.26 mg Se/kg DM) that was harvested from sodium-selenite-fertilized lands (22.5, 45.0, or 89.9 g Se/ha, respectively) during the backgrounding phase had considerably lower BRD mortality and greater growth performance in the feedlot stage in a dose-dependent manner compared with control calves receiving unfertilized alfalfa hay (0.07 mg Se/kg DM). Moreover, calves supplemented with high Se-yeast before weaning had enhanced in vitro macrophage phagocytosis on day 22 post-weaning compared with unsupplemented control calves [14]. Similarly, another report showed higher natural killer cell cytotoxicity in bulls that received selenium yeast and vitamin E compared with control bulls [23]. The positive effects of Se supplementation may be associated with an enhancement of the antioxidant capacity and cholesterol metabolism [25], upregulation of the mitochondrial gene expression capacity, and downregulation of oxidative stress-related genes, as previously demonstrated [15]. More recent studies showed that feeding Se-biofortified alfalfa hay to weaned beef calves prior to entering the feedlot enhanced nasopharyngeal microbiome diversity, which may explain in part the benefits of high-Se diets for prevention of BRD in beef calves [20,21].

Nevertheless, other trials have shown no effects of Se supplementation on beef cattle immune and health parameters [90,94,122]. Supplementation with Se and vitamin E in pre-weaning Hereford/Angus calves with normal mineral and vitamin status did not affect the pre- or post-weaning weight gain or the immune response to BVDV and BHV1 following vaccination [94]. Studies aimed to evaluate the effects of Se and Vitamin E supplementation on the immune response, health and performance of feedlot steers showed an increase in antibody production against *P. haemolytica*, but did not demonstrate positive effects improving health status [90]. Intriguingly, Reffett-Stabel et al. [122] reported that weaned calves that had been nursed by cows receiving Se-adequate diet had lower antibody titers after challenge with *P. haemolytica* than calves nursed by cows receiving Se-deficient diet.

Despite Se being the most deficient TM in forage-based diets [83,85] and Se supplementation providing multiple known benefits, caution should be taken when supplementing beef cattle with Se to ensure the provision of adequate amounts without reaching toxic levels [123].

### 6.4. Oral Manganese Supplementation

According to NASEM [82], finishing steers and beef cows require 20 and 40 mg Mn /kg DM, respectively [82]. There is limited evidence of the effects of individual Mn supplementation on the immunity and health of beef cattle. A study was performed to evaluate the effects of different dietary Mn levels (10, 30, or 50 mg of supplemental Mn/kg DM from MnSO) on growth and reproduction of Angus and Simmental heifers. Heifers were fed a diet containing cottonseed hulls, corn gluten feed, citrus pulp, and ground corn, and the control diet contained 15.8 mg of Mn/kg DM. Mn supplementation did not affect reproductive performance, indicating that 15.8 mg of Mn/kg DM is adequate for growth and fertility of heifers [101].

### 6.5. Oral Chromium Supplementation

The Cr requirements for beef cattle have not been established [82]. The positive effects of oral Cr supplementation on health and performance of beef steers have been previously demonstrated [7,16]. Improvement in growth performance and lower BRD morbidity have been reported in stressed newly-received cattle supplemented with Cr [7,16]. In the initial study using calves supplemented with either 0 (controls) or 0.4 mg/kg/day of Cr (from high-Cr yeast) for 28 days revealed that Cr supplementation significantly increased ADG (30%) and feed efficiency (27%), suggesting a possible Cr deficiency in the experimental calves [7]. The antibody levels were greater for the Cr-supplemented calves, with no effect on morbidity [7]. Another experiment during a 70-day growth period using the control calves from the previous study did not show significant effects of Cr supplementation on ADG or feed efficiency, but it was associated with reduction of blood glucose and cortisol, indicating that Cr might positively influence the immune system of stressed animals. These two studies suggested that timing of Cr supplementation in relation to stress and pathogen exposure should be considered [7].

A subsequent study showed that lower level of Cr supplementation (0.2 mg/kg) was able to improve ADG and reduced morbidity in newly-received stressed beef calves compared with calves receiving higher Cr supplementation (0.5 and 1 mg/kg) and no supplementation [124]. This study confirmed the results of the previous reports on the effects of Cr supplementation on growth performance, immuno-competence and reduction of cortisol levels [124]. A successive trial reported significant reduction of morbidity when supplementing calves with an amino acid chelated Cr (15%) compared to high-Cr yeast (33%) and non-supplemented control (55%) [125]. Further research testing the effects of Cr supplementation on the immune response elicited by a MLV vaccine containing BHV1 and PI_3_V in stressed calves showed that Cr supplementation had significant effects on BHV1 specific antibody response. The results showed that 75% of the BHV1 seropositive calves were from the Cr-supplemented group, whereas only 25% were from the control group [79]. Further, Cr supplementation has been documented to also affect the cell-mediated immunity of beef cattle. A greater local cell-mediated immune response after intradermal injection of phyto-hemagglutinin was observed in Angus and Angus crossbred steers receiving ad libitum supplementation with high-Cr yeast than control steers or those supplemented with CrCl3 or Cr nicotinic acid. In addition, animals supplemented with Cr-nicotinic acid complex in corn silage-based diets had a greater blastogenic response to the same antigen than steers supplemented with CrCl3 [126].

## 7. Single-Use, Pulse-Dose Administration of Trace Minerals

Oral free-choice TM supplementation may lead to mineral deficiencies due to high variability in mineral composition and intake, mineral binding to undigested feed particles, reduced absorption in the digestive tract, and antagonisms. Strategic extra supplementation using single-administration, pulse-dose trace mineral products (oral or injected) is becoming very popular on beef farms to prevent TM deficiencies or improve the TM status. This provides the required TM amounts during critical points of the production cycle, when the animals have greater TM demands (e.g., before transportation or at vaccination). However, this strategic supplementation is not intended to substitute the regular oral mineral supply.

Oral single pulse TM supplementation (e.g., drench, paste or bolus) is being used in some beef cattle farms. However, there is not enough evidence of the effects of these products on the animals’ TM status [127]. A study performed by Dr. Hansen’s team at Iowa State University demonstrated that oral single, pulse-dose trace mineral supplements (paste or drench) did not efficiently increase blood or liver trace mineral concentrations in Angus-cross steers with adequate TM status [127]. Administration of injectable trace minerals (ITM) resulted in faster and greater increase in Zn, Se and Mn concentrations (through 24 h) compared with other single-dose supplementation treatments (drench, paste and boluses) [127]. In addition, hepatic Se levels were also higher (through day 29) in the steers treated with ITM compared to the other treatment groups. However, ruminal boluses (Reloader 250™ bolus) generated greater long-term hepatic Se concentration than ITM (on day 91) and the other products (on day 120). The choice of the single-dose supplementation method depends on whether fast effects (e.g., ITM concurrent with vaccination) or a long-term, sustained-release TM supplementation (e.g., Se rumen bolus) are needed to support a particular management strategy [127].

## 8. Injectable Trace Minerals on Beef Cattle Immunity and Health

The use of ITM formulations (containing Se, Cu, Zn and Mn) has several advantages for beef production systems. It provides a known and well-controlled quantity of TM that is rapidly and efficiently absorbed and stored after injection [128]. This is important for cattle having reduced DMI and mineral consumption (e.g., at the time of weaning and vaccination or during periods of transportation and receiving) or limited access to free- choice mineral supplements (e.g., extensive rangeland systems, flooded pastures, etc). Therefore, administration of ITM decreases the variability in TM status observed in animals with free choice mineral intake [129]. A commercially available ITM product containing Zn (60 mg/mL), Cu (15 mg/mL), Mn (10 mg/mL) and Se (5 mg/mL; Multimin^®^-90. Multimin^®^, Axiota^®^ Animal Health) has been proven to be absorbed quickly, increasing blood TM levels within 8 to 10 h after injection and followed by storage in liver at approximately 24 h post injection [128,130]. Administration of this compound results in an increase in enzymatic (e.g., GPx and SOD) activity by 24 h following administration, with a maximum enzymatic function by 15 days post injection. These effects may persist for approximately 90 days [128,130,131].

Studies performed by the author’s research team (GRAVID™) at the University of Georgia [30,132,133,134,135,136,137] and others [138,139,140] have demonstrated the effects of the administration of ITM (containing Cu, Se, Zn, and Mn) on the protective immunity elicited by vaccination against BRD and neonatal calf diarrhea. Arthington and Havenga [139] reported that administration of ITM concurrently with BRD MLV vaccination increased and/or mitigated the decrease of trace mineral levels (Cu, Se, and Zn) after vaccination of beef calves. In that study, treatment with ITM enhanced the production of BHV1-specific serum neutralizing antibodies on days 14, 30, and 60 post-vaccination. Similarly, administration of ITM concurrently with a BRD MLV vaccine + *M. haemolytica* and *P. multocida* bacterin in weaned Holstein bull calves with normal TM status resulted in increased hepatic Se, Cu and Mn concentrations. In addition, ITM calves had greater antibody titers (26.7% more calves having seroconversion) to BVDV1 on day 28 after primary vaccination compared with the control group [132]. In the same study, calves treated with ITM showed an earlier and stronger mononuclear leukocyte proliferation to BVDV1 and BRSV following vaccination compared with the control group. Moreover, ITM administration also enhanced antibody production and leukocyte proliferation against *M. haemolytica* and *P. multocida,* respectively [134]. The observed decrease in TM concentration in plasma [139] and liver [132] after vaccination in the untreated control calves is believed to be associated with TM utilization during the development of the immune response. Therefore, strategic supplementation with ITM at the time of vaccination may be a critical control point in regular beef cattle vaccination programs. Studies performed by Bittar et al. [135] and Roberts et al. [140] using newly-received beef calves demonstrated that treatment with ITM concomitantly with MLV parenteral vaccination induced faster and stronger BVDV-specific antibody response [135,140]. A rapid and robust antibody response against BRD pathogens might be of significant value by conferring earlier protection following vaccination in newly-received, highly stressed cattle, which are at high risk of suffering from BRD. Our study indicated that ITM administration mitigated the decrease in CD4^+^ and CD8^+^ T cells associated with BVDV2 acute infection in calves that were BVDV2-challenged five days after arrival and immunization [135]. These results support our previous findings of greater leukocyte proliferation in ITM-treated calves [132]. Further studies in our lab also demonstrated that ITM supplementation at the time of intranasal (IN) vaccination of dairy bull calves controlled the CD8^+^ T cell reduction after BVDV2 + BHV1 challenge [136]. In addition, calves treated with ITM at the time of IN vaccination had reduced inflammation and damage of the upper respiratory tract mucosa after BVDV2 + BHV1 challenge compared to vaccinated, saline-treated calves [137].

In another challenge trial, the calves treated with ITM at the time of vaccination had increased ADG (during the first 14 days) following a BVDV2 challenge given five months after vaccination. Further, ITM-treated calves had greater platelet counts compared with the control calves [133]. A more recent study examined the effects of a different injectable solution containing Cu and Zn on weight gain, hematological parameters, and immune response of pre-weaning beef calves [141]. Supplementation with ITM improved ADG and the antibody response to inactivated BHV1 vaccination [141].

The effects of ITM on growth performance and feed efficiency of cattle have been variable. Arthington et al. [142] performed three experiments to assess the effects of ITM (Multimin^®^-90) on health and performance of pre- and post-weaned Brangus-crossbred beef calves. Administration of ITM improved Cu, Se, and Zn status and acute phase proteins, but did not improve growth performance of pre-weaned and post-weaned calves. However, at the subsequent developmental stage (peri-pubertal period), the heifers treated with ITM had enhanced ADG, antibody titers to porcine red blood cells, and liver Se concentrations compared with control heifers [142]. Genther and Hansen [130] evaluated the growth performance and meat quality of beef cattle treated with ITM. Supplementation with ITM improved growth of mildly TM-deficient steers and improved rib eye area and marbling score regardless of initial TM status.

The use of another ITM formulation containing 10 mg/mL Cu, 10 mg/mL Mn, 5 mg/mL Se, 40 mg/mL Zn (Adaptador Min, Biogénesis Bago, Buenos Aires, Argentina) administered in conjunction with vitamins A, and E decreased body weight loss in the steers submitted to 26-day preconditioning from weaning to transport [143]. In contrast, other studies to evaluate the efficacy of ITM at the time of weaning in Nellore calves showed no effect of mineral supplementation on growth performance, even though it caused an increase in plasma concentrations of antioxidant enzymes (SOD and GPx) [144].

The effects of ITM supplementation on *Bovine coronavirus* antibody response and passive transfer after vaccination against neonatal calf diarrhea in beef cattle have been recently evaluated [30]. Administration of ITM improved Se and Cu status and enhanced colostrum *Bovine coronavirus* antibody response to vaccination in beef cows with marginal Se, Cu and Zn deficiencies. In that study, calves that had nursed ITM-treated cows had enhanced *Bovine coronavirus* antibody passive transfer [30].

The research-based evidence indicates that strategic administration of ITM containing Se, Cu, Zn and Mn in calves receiving free-choice oral TM supplementation improves the oxidative status and the immune response and protection elicited by IN or parenteral (attenuated or inactivated) vaccination against bovine respiratory pathogens. In addition, there is limited evidence that under certain conditions (depending on TM status, developmental state, weaning management, among others) ITM supplementation may positively affect cattle growth performance.

## 9. Conclusions

Trace mineral supplementation is an essential component of beef cattle health programs. Micro-minerals such as Se, Cu, Zn, Mn and Cr play significant roles in numerous metabolic pathways, homeostatic functions, oxidative balance and immune response. Therefore, cattle TM status has a direct impact on health and performance. Adequate TM supplementation is crucial to maintaining an optimal TM status, especially during episodes of stress and oxidative imbalance (e.g., during transportation) or higher metabolic and immune demands (e.g., at vaccination). Oral TM supplementation is a common and highly recommended practice for beef cattle operations, as numerous reports demonstrate its benefits on immunity, health and performance. However, due to the high variation in research experimental conditions and TM formulations, interpretation and comparison of data on the effects of oral TM supplementation on animal health is difficult. In addition, clinical and experimental evidence indicates that there is an enormous source of variation in TM bioavailability in animals receiving oral TM. It is highly recommended to evaluate the herd’s mineral profile and the amount of TM that the animals may be consuming to determine the TM supplementation regime. Research studies and the author’s field experience support the practice of strategic ITM supplementation (without replacing traditional oral mineral supplementation) to enhance TM and oxidative status, overall cattle health and the immune response prior to challenging episodes of stress or following vaccination. This may result in decreased cattle morbidity and costs of treating sick animals, which would positively impact the productivity of beef cattle systems.

## Figures and Tables

**Table 1:** Reference values of trace mineral requirements, maximum tolerable concentrations and hepatic concentrations in adults and growing calves.

	^†^ Requirements Range mg/kg DM	^†^ Maximum Tolerable Concentration mg/kg DM	Hepatic Concentrations Mean (Range *) μg/g or Ppm
Selenium	0.10	5.0	0.8–1.0 (0.7–2.5)
Copper	10	40	25–50 (50–600)
Zinc	30	500	80–100 (90–400)
Manganese	40	1000	9 (5–15)

^†^ Nutrient Requirements of Beef Cattle, 8th ed. National Academy Press: Washington, DC, USA, 2016 [82]. * Values are expressed on a dry tissue basis. Reference ranges of hepatic TM concentrations as reported by Herdt and Hoff [81]. DM: dry matter.

## Data Availability

Not applicable.
